# The genus *Gallerucida* Motschulsky in Taiwan (Insecta, Coleoptera, Chrysomelidae, Galerucinae)

**DOI:** 10.3897/zookeys.723.21545

**Published:** 2017-12-18

**Authors:** Chi-Feng Lee

**Affiliations:** 1 Applied Zoology Division, Taiwan Agricultural Research Institute, 189 Chung-Cheng Road, Wufeng, Taichung 413, Taiwan

**Keywords:** Host plants, leaf beetles, Polygonaceae, taxonomic revision, Vitaceae

## Abstract

Species within the genus *Gallerucida* Motschulsky recorded in Taiwan are revised. *Gallerucida bifasciata* Motschulsky 1861 *G. lutea* Gressitt & Kimoto 1963 *G. sauteri* Chûjô 1938 and *G. shirozui* Kimoto 1969 are redescribed. *Sphenoraia chujoi* Lee 2014 is proposed as a junior synonym of *G. flaviventris* (Baly 1861). *Gallerucida thoracica* (Jacoby 1888) is recorded as new for Taiwan and redescribed. Lectotypes are designated for *Gallerucida nigrita* Chûjô 1935 *G. sauteri* Chûjô 1938 and *Eustetha thoracica* Jacoby 1888. Biological notes are given on all Taiwanese species of *Gallerucida*.

## Introduction

The genus *Gallerucida* Motschulsky, 1861 is widespread in the Oriental and East Palaearctic regions, with highest species diversity in China. Of 66 valid species that were recognized by Wilcox (1971), 43 species were recorded from China. A number of new and newly recorded species have been added recently to the Chinese fauna (Chen 1992, Yang 1994a, 1994b, 1997). Currently, 60 species are recognized from China (Yang et al. 2015). In addition, one additional species, *Galluercida gebieni* Weise, 1922 should be added to the Chinese fauna since it was removed from synonymy with *G. singularis* Harold, 1880 by Lee and Bezděk (2013).

Adults within the genera *Gallerucida* Motschulsky, 1861 and *Laphris* Baly, 1864 are easily recognized by their projecting anterior metasterna that cover most of the mesosterna. *Gallerucida* adults can be separated from those of *Laphris* by the comparatively shorter antennomeres III (subequal or twice length of antennomeres II; by contrast antennomeres III are four times the lengths of II in *Laphris*). Adults of the genus *Sphenoraia* Clark, 1865 also look like those of *Gallerucida* and *Laphris*, but they can be distinguished easily by the absence of the projecting anterior process of the metasternum. *Gallerucida nigromaculata* (Baly, 1861) and *G. singularis* Harold, 1880 were firstly recorded from Taiwan by Chûjô (1935) together with description of a new species, *G. nigrita* (*G. nigromaculata* and *G. nigrita* were synonymized with *G. bifasciata* Motschulsky). Chûjô (1938) described a new species, *G. sauteri* Chûjô. Kimoto (1969) recorded *G. flaviventris* (Baly, 1861) and *G. lutea* Gressitt & Kimoto, 1963 for the first time and described a new species, *G. shirozui* Kimoto. Takizawa (1978) described a new species, *G. quadraticollis* Takizawa which was synonymized with *G. sauteri* Chûjô. Lee and Bezděk (2013) first listed *G. gebieni* Weise, 1922 from Kimen and Nankan islands. This brings the total to seven species reported from Taiwan to date.

*Gallerucida bifasciata* Motschulsky is an abundant species that is considered as biological control agent for invasive species of Polygonaceae (Ding et al. 2004; Wang et al. 2008; Wang et al. 2010). However, others are little-known except for scattered original taxonomic descriptions.

The Taiwan Chrysomelid Research Team (TCRT) was founded in 2005 and is composed of 10 members. All of them are amateurs interested in producing a complete inventory of Chrysomelid species in Taiwan. Members of the genus *Gallerucida* have been collected and studied, and host plants recorded. Life histories for almost all species were documented by laboratory rearing. The results of these efforts are the subject of the current paper.

## Materials and methods

For rearing studies, larvae were placed in small glass containers (diameter 142 mm × height 50 mm) with cuttings from their host plants. When mature larvae began searching for pupation sites, they were transferred to smaller plastic containers (diameter 90 mm × height 57 mm) filled with moist soil (about 80% of container volume).

For taxonomic study, the abdomens of adults were separated from the fore body and boiled in 10 % KOH solution, followed by washing in distilled water to prepare genitalia for illustrations. The genitalia were then dissected from the abdomen, mounted on slides in glycerin, and studied and drawn using a Leica M165 stereomicroscope. For detailed examinations a Nikon ECLIPSE 50i microscope was used.

At least three pairs from each species were examined to delimit variability of diagnostic characters. For species collected from more than one locality, at least one pair from each locality was examined. Length was measured from the anterior margin of the eye to the elytral apex, and width at the greatest width of the elytra.

Specimens studied herein are deposited at the following institutes and collections:

**BMNH** The Natural History Museum, London, UK [Michael Geiser];

**BPBM** Bernice P. Bishop Museum, Hawaii, USA [James Boone];

**CAS** California Academy of Sciences, California, USA [David H. Kavanaugh];

**EIHU** Systematic Entomology, The Hokkaido University Museum, Sapporo, Japan [Masahiro Ôhara];

**EUMJ** Ehime University, Matsuyama, Japan [Hiroyuki Yoshitomi];

**JBCB** Jan Bezděk collection, Brno, Czech Republic;

**KMNH** Kitakyushu Museum of Natural History and Human History, Kitakyushu, Japan [Yûsuke Minoshima];

**KUEC** Faculty of Agriculture, Kyushu University, Fukuoka, Japan [Osamu Tadauchi];

**MCZC** Museum of Comparative Zoology, Harvard University, Massachusetts, USA [Philip D. Perkins];

**KUEC** Museum National d’Histoire naturelle, Paris, France;

**NHMB** General collection, Naturhistorisches Museum, Basel, Switzerland [Matthias Borer];

**NMNS** National Museum of Natural Science, Taichung, Taiwan [Ming-Luen Jeng];

**SDEI** Senckenberg Deutsches Entomologisches Institut, Müncheberg, Germany [Konstantin Nadein]

**TARI** Taiwan Agricultural Research Institute, Taichung, Taiwan.

Exact label data are cited for all type specimens of described species; a double slash (//) divides the data on different labels and a single slash (/) divides the data in different rows. Other comments and remarks are in square brackets: [p] – preceding data are printed, [h] – preceding data are handwritten, [w] – white label, [y] – yellow label, [g] – green label, [b] – blue label, and [r] – red label.

## Taxonomy

### Gallerucida bifasciata

Taxon classificationAnimaliaORDOFAMILIA

Motschulsky

Figs 1 , 2 , 3

Gallerucida bifasciata Motschulsky, 1861: 24 (Japan); Solsky 1872: 259 (East Siberia); Chûjô 1940: 6 (Korea); Chûjô 1941: 160 (Korea); Gressitt and Kimoto 1963: 721 (China: Jilin, Shaanxi, Gansu, Sichuan, Hubei, Guizhou, Jiangxi, Fujian, Zhejiang, Jiangsu); Kimoto 1965a: 399 (infraspecific variation between north and south Japan); Kimoto and Hiura 1965: 38 (Japan); Kimoto 1966: 34 (Taiwan); Kimoto and Kawase 1966: 47 (China: Jilin); Kimoto 1969: 68 (Taiwan); Wilcox 1971: 201 (catalogue); Kimoto 1989: 260 (Taiwan); Kimoto 1991: 17 (Taiwan); Kimoto and Chu 1996: 92 (catalogue); Kimoto and Takizawa 1997: 392 (catalogue); Lee and Cheng 2007: 104 (biology); Beenen 2010: 459 (catalogue); Yang et al. 2015: 171 (catalogue).
Galerucida [sic!] bifasciata: Weise 1886: 578 (Amur); Heyden 1887: 263 (Korea); Weise 1924: 140 (catalogue); Ogloblin 1936: 354 (redescription); Kimoto 1965b: 488 (Taiwan).
Melospila bifasciata : Baly 1874: 185.
Melospila nigromaculata Baly, 1861: 297; Harold 1876: 3591 (as synonym of G. bicolor, synonymy confirmed).
Gallerucida nigromaculata : Chûjô & Kimoto 1961: 163 (host plants); Chûjô 1962: 154 (redescription).
Galerucida [sic!] nigromaculata: Weise 1922: 92 (China: Fujian); Chûjô 1935: 169 (Taiwan); Ogloblin, 1936: 356 (redescripton).
Gallerucida bifasciata nigromaculata : Takizawa 1980: 73 (Korea); Takizawa 1985: 10 (as synonym of G. bifasciata).
Galerucida [sic!] nigrofasciata Baly, 1879: 453 (should be error for G. nigromaculata Baly because G. nigromaculata is the only one of Baly’s species which is treated by Harold (1876) as a synonym of G. bicolor) (as synonym of G. bifasciata, synonymy confirmed).
Melospila consociata Baly, 1874: 185; Ogloblin 1936: 354 (as synonym of G. bifasciata, synonymy confirmed).
Galerucida [sic!] nigrita Chûjô, 1935: 168; Chûjô 1962: 153 (redescription); Kimoto 1966: 34 (as synonym of G. bifasciata, synonymy confirmed).

#### Type material.

*Gallerucida bifasciata*. Lectotype ♂ (KUEC), here designated, labeled: “Galerucida / bifasciata / Motch. / Type / Japonia [h, w] // Ex-Musæo / E. Harold [p, w]”. Number of paralectotypes is uncertain.

*Melospila nigromaculata*. Lectotype ♂ (BMNH), here designated, labeled: “Galerucida / nigromaculata / Baly / N. China [h, g] // Type [p, w, circular label with red border] // Type [h, w] // Baly Coll. [p, w]”. Number of paralectotypes is uncertain.

*Melospila consociata*. Lecotype ♀ (BMNH), here designated, labeled: “Hakodate / Mr. Moor [h, w, with pencil written on the back of the label which specimen glue on] // Hakodate [p, w] // Japan. / G. Lewis, / 1910—320. [p, w]”. Number of paralectotypes is uncertain.

*Glaerucida nigrita*. Lectotype ♂ (TARI), here designated, labeled: “Formosa. / Musha [= Wushe, 霧社], 1919. / V 18 – VI 15. / T. Okuni, [p, w] // CO / Types [p, w, yellow letters, circular label with yellow border] // *Galerucida* / *nigrita* Chûjô [h] / DET. M. CHUJO [p, g] // 1928 [p, w]”. Paralectotypes. 2♂♂, 1♀ (TARI), same as lectotype but with “2183, or 2184, or 1929; 1♂ (TARI): “Horisha / Apr. 2, 1919 [h, w] // CO / Types [p, w, yellow letters, circular label with yellow border] // *Galerucida* / *nigrita* Chûjô [h] / DET. M. CHUJO [p, g]” // 2182 [p, w]”; 1♂ (SDEI): “Taihorinsho [= Talin, 大林] / Formosa / Sauter [p] VIII. [h] 07.09 [p, w] // Syntypus [p, r] // *Galerucida* / *nigrita* Chûjô [h] / DET. M. CHUJO [p, g] // DEI Müncheberg / Col-09171 [p, g]”.

#### Diagnosis.

*Gallerucida bifasciata* adults are easily recognized by their black bodies, with or without yellowish brown stripes, and strongly serrate antennae. Aedeagi of male endophallic sclerite complex is characterized by its short endophallic sclerite complex, and the median sclerite is similar to the lateral sclerite in length. By contrast, the endophallic sclerite complex is comparatively longer, and the median sclerite is much longer than the lateral sclerite in other species.

#### Redescription.

**Figures 1.**
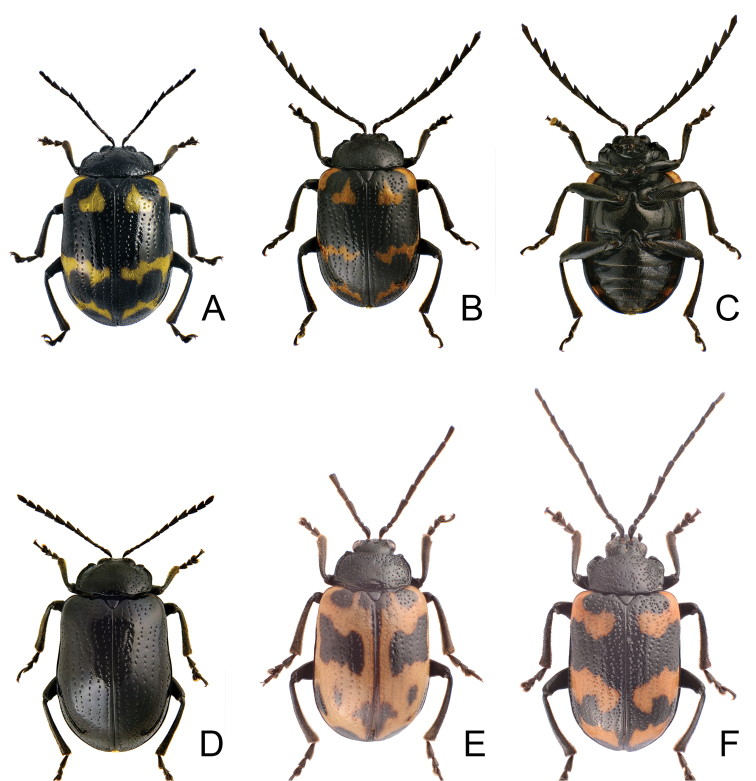
Habitus of *Gallerucida bifasciata* Motschulsky. **A** Female, dorsal view **B** Male, color variation, ventral view **C** Ditto, ventral view **D** Female, stripes completely reduced, dorsal view **E** Male, stripes well developed, dorsal view **F** Male, from northern Japan.

**Figures 2.**
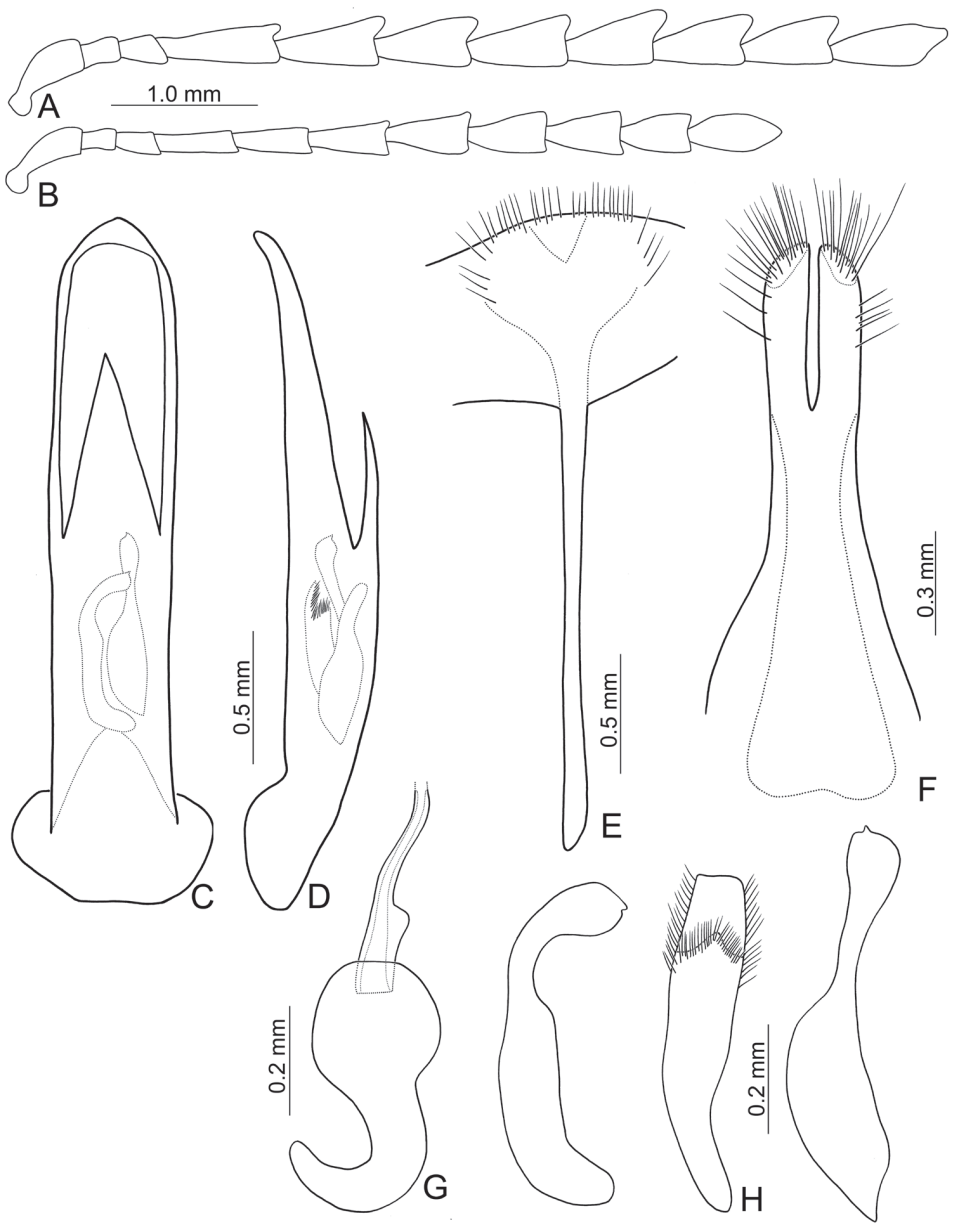
Diagnostic characters of *Gallerucida bifasciata* Motschulsky. **A** Antenna, male **B** Antenna, female **C** Penis, dorsal view **D** Penis, lateral view **E** Abdominal ventrite VIII **F** Gonocoxae **G** Spermatheca **H** Endophallic sclerites.

Length 7.1–11.2 mm, width 4.2–6.0 mm. General color (Fig. 1A–C) black; elytra with three pairs of transverse, yellowish brown or orange stripes, one pair at baso-lateral angles curved inwards; second pair behind middle sinuate, expanding posteriorly at 1/3 and 2/3 distance between suture and lateral margins; third pair near apex curved inwards, expanding anteriorly at 1/3 and 2/3 distance between suture and lateral margins; lateral margin of abdomen yellowish brown. Antenna serrate in males (Fig. 2A), length ratios of antennomeres I–XI 1.0 : 0.4 : 0.5 : 1.3 : 1.2 : 1.1 : 1.1 : 1.1 : 1.1 : 1.1 : 1.3, length to width ratios of antennomeres I–IX 2.6 : 1.4 : 1.5 : 3.3 : 2.3 : 2.2 : 2.1 : 1.9 : 2.2 : 2.1 : 2.9; less serrate and shorter in females (Fig. 2B), length ratios of antennomeres I–XI 1.0 : 0.4 : 0.5 : 0.9 : 0.9 : 0.9 : 0.9 : 0.9 : 0.9 : 0.8 : 1.1, length to width ratios of antennomeres I–IX 3.1 : 1.6 : 2.0 : 3.5 : 3.1 : 2.5 : 2.0 : 1.9 : 1.8 : 1.9 : 2.5. Pronotum transverse, 1.9× wider than long, disc convex, with indistinct depressions at sides, disc with microreculation, and extremely coarse, sparse punctures, and minute, sparse punctures between coarse punctures; lateral margin rounded; apical margin concave; basal margin convex. Elytra parallel-sided; 1.4× longer than wide, disc without micro-reticulation but with extremely coarse punctures arranged into striae, with minute punctures between coarse punctures. Penis (Fig. 2C, D) elongate, 5.8× longer than wide; parallel-sided; apex lanceolate; slightly curved in lateral view; ventral surface well sclerotized; endophallic sclerite complex (Fig. 2H) small, about 0.3× as long as penis, composed of one median sclerite and one pair of lateral sclerites, median sclerite longitudinal, with dorsal process at apical 1/4, with dense setae along apical margin of dorsal process; lateral sclerites longitudinal and slightly longer than median, about 1.2× median sclerite, asymmetric, curved near apex, apices circular and with one acute tooth. Gonocoxae (Fig. 2F) elongate, connected from base to apical 1/3, apices rounded, with dense elongate setae; base shallow bifurcate. Ventrite VIII (Fig. 2E) longitudinal, apex transverse, apical margin truncate; with dense short setae along apical margin; spiculum extremely slender. Receptacle of spermatheca (Fig. 2G) strongly swollen; pump short but strongly curved; proximal spermathecal duct wide and deeply inserted into receptacle.

#### Variations.

Kimoto (1965a) noted that specimens (Fig. 1F) collected from Hokkaido and Northeast Honshu possess coarser punctures on the pronotum and elytra, and reticulate microsculpture on the pronotum, and treated them as *G. bifasciata* and *G. consociata*. Some individuals from North China possess the well-developed yellowish brown stripes on the elytra with several black spots (Fig. 1E). By contrast, some specimens from Taiwan have the yellowish brown stripes completely reduced (Fig. 1D) and were identified as *G. nigrita*.

#### Host plants.

Polygonaceae: Fallopia multiflora var. hypoleucum (Ohwi) Yonek. et H. Ohashi (present study); *F. sachaliensis* (F. Schmidt) Ronse Decr. (=*Polygonum sachaliense* and *Reynoutria sachalinensis*) (Chûjô and Kimoto 1961); *Persicaria perfoliata* (L.) H. Gross (Lee and Cho 2006); *Polygonum cuspidatum* Sieb. & Zucc. (= *Reynoutria japonica* and *Fallopia japonica*) (Chûjô and Kimoto 1961); *Rheum undulatum* Linn. (Lee and Cho 2006); *Rumex acetosa* Linn.; *Ru. japonicus* Houtt. (Chûjô and Kimoto 1961); *Ru. aquaticus* Linn.; *Ru. crispus* Linn. (Lee and Cho 2006). Its host specificity was examined by Wang et al. (2008). Adults strongly preferred *Fallopia japonica* (=*Polygonum cuspidatum*), *Persicaria perfoliata*, and *Polygonum multiflorum* (=*Fallopia multiflora*).

#### Biology.

**Figures 3.**
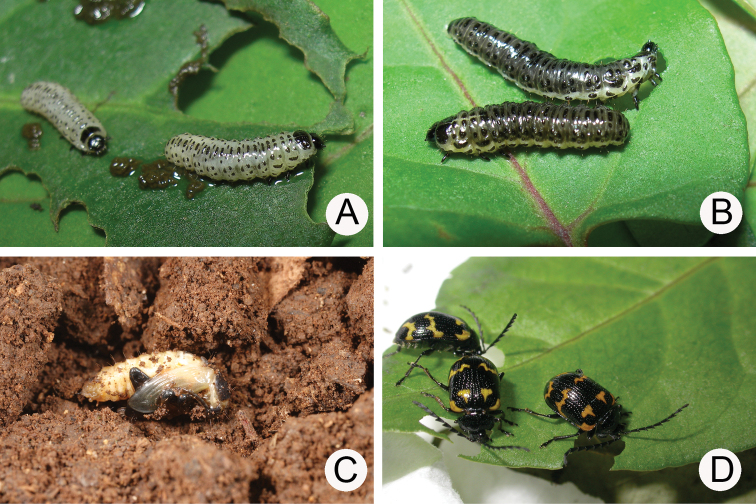
Field photographs of *Gallerucida bifasciata* Motschulsky. **A** Early instar larvae **B** Mature larvae **C** Pupa **D** Adults.

*Gallerucida bifasciata* populations are presumably multivoltine. The following life cycle information is based on our (TCRT) observations (Lee and Cheng 2007). Females began to deposit an average of 20 eggs in single egg masses during mid-January. Eggs hatched in 11–14 days. The larvae (Fig. 3A) fed on leaves and the larval duration was 14–15 days. Mature larvae (Fig. 3B) burrowed into the soil and built underground chambers for pupation. Duration of the pupal stage (Fig. 3C) was 14–19 days. Newly emerged adults appeared during spring and were active (Fig. 3D) during summer and autumn.

#### Other material examined.

**CHINA.** Anhui: 2♂♂, 1♀ (NHMB), Dabieshan [大別山], 21-24.VI.1998, leg. Bolm; Fujian: 1♀ (BMNH), Wuyishan [武夷山], Jianyang [建陽], 27.III.1980, leg. S. Q. Jiang; 2♂♂ (BPBM), Shui-Pei-Kai, Shaowu, 26.III.1942, leg. T. C. Maa; Guanxi: 1♂ (TARI), Dayaoshan [大瑤山], 14.V.2016; Hebei: 2♂♂, 3♀♀ (NHMB), Wudanshan [武當山], 5-7.VII.1998, leg. Bolm; 1♂, 1♀ (JBCB), Xintai [邢台], Taihang mts. [太行山], Neiqiu [內丘], 8-11.VI.2004, leg. M. Knížek; 2♂♂ (JBCB), border between Hebei and Inner Mongolian, road Chengda-Chifeng, pass 1600 m, 1-2.VI.2000; Heilongjiang: 1♂ (BMNH), Erlungshan [二龍山], 29.V.1966, leg. P. M. Hammond; 1♀ (BMNH), Harbin [哈爾濱], 29.VI.1952; Hubei: 1♀ (BMNH), Ichang [= Yichang, 宜昌], B.M. 1922-212, leg. C. T. Bowring; 1♂ (BPBM), Trail between Mo-Tai-Chi and Sang-Hou-Ken, 19.VII.1948, leg. Gressitt & Djou; Jiangsu: 1♂ (BMNH), Nanjing [南京], 1935, coll. IZAS; Shaanxi: 1♂ (BPBM), Mts. Chin-Ling [秦嶺山], IV.-V.1904; 1♀ (BMNH), Cuihuashan [翠華山], 19.IX.1980, leg. P. M. Hammond; 1♂ (BMNH), Huashan [華山], 30.VII.1966, leg. P. M. Hammond; 3♂♂, 1♀ (JBCB), same locality, 17-22.VI.1991, leg. Z. Kejval; Sichuan: 2♂♂ (TARI), Bayueshan [巴岳山], 21.IV.2013; 1♂ (TARI), Fenghuang [鳳凰鎮], 30.III.2013; 1♂ (NHMB), Guanxian [灌縣], 27.VI.1990, leg. L. & M. Bocák; Zhejiang: 1♂ (BPBM), Hangchow [=Hangzhou, 杭州], 11.VI.1924, leg. J. F. Illingworth; 1♀ (BMNH), same locality, 8.IV.1930, leg. P. H. Tsai; **JAPAN.** Hokkaido: 3♂♂, 1♀ (JBCB), Sapporo, Oshoro, 15.VI.1997, leg. V. Košťál; Honshu: 2♀♀ (JBCB), Aomori, Fukaura, 11-13.VI.1999, leg. M. Hayashi; 1♂ (BMNH), Fukushima, 26-29.VII.1881, coll. G. Lewis; 1♂, 1♀ (NHMB), Mt. Fuji, 200 m, 4-13.VIII.1985, leg. G. J. Minet; 1♂ (NMNS), Gifu, Kamagatani, 7.VII.1946, leg. T. Takahashi; 1♀ (NMNS), Gifu, Suhara, 3.VI.1956, leg. K. Ohbayashi; 1♀ (NMNS), same but with “26.V.1957”; 16♂♂, 2♀♀ (NMNS), Hyogo, Mt. Oginosen, 4.V.1964, leg. M. H. Chûjô; 27♂♂, 2♀♀ (NMNS), same locality, 1-5.V.1965, leg. Y. Ohira; 1♂, 1♀ (BMNH), Kyoto, Kibune, V.1951, leg. A. Nobuchi; 3♂♂ (BMNH), Nikko, 3-21.VI.1880, coll. G. Lewis; 2♂♂, 1♀ (BMNH), Nikko dist., Kozawa, 15.VIII.1980, leg. P. M. Hammond; Kyushu: 1♂ (TARI), Fukuoka, Mt. Inunaki, 5.V.1939, leg. S. Nisiguti; 2♀♀ (TARI), same but with “19.V.1940”; 1♀ (TARI), same but with “26.V.1940”; 1♂ (BMNH), Nagasaki, coll. G. Lewis, 1910—320; **SOUTH KOREA**. 2♀♀ (JBCB), Chungcheongbuk-do, Daegang-myeon, Danyang-gun, 12.VI.2008, leg. J. M. Kwon; 1♂ (JBCB), Gyeongsangbuk-do, Cheongsong-gun, Hyeonseo-myeon, Sachon-ri, 5.VI.2010, leg. H. W. Cho; 1♂ (NHMB), Kyongju National Park, VIII.1979, leg. G. M. Récolt; **RUSSIA**. 2♂♂, 1♀ (JBCB), Primorskij kraj, Arsenev, VI.1991, leg. Štrba; 1♂ (JBCB), Primor’ye, Lazo, VII.1990, leg. S. Pokorný; **TAIWAN**. Hsinchu: 5♀♀ (TARI), Kuanhsi [關西], 9.II.2007, leg. H.-H. Han; Hualien: 3♂♂, 2♀♀ (TARI), Fuli [富里] – Tungho [東河] (in Taitung), 9-11.XI.1982, leg. K. C. Chou & S. P. Huang; Kaoshiang: 1♀ (TARI), Hsiaokuanshan [小關山], 15.V.2016, leg. B.-X. Guo; 1♀ (TARI), Shanping [扇平], 7.VI.2014, leg. W.-C. Liao; 1♀ (TARI), Taoyuan [桃源], 15.IV.2013, leg. L.-P. Hsu; 1♂ (NMNS), Tengchih [藤枝], 22.VIII.1996, leg. M.-L. Chan; 2♂♂ (TARI), same locality 28.III.2015, leg. W.-C. Liao; 1♂ (TARI), Tona trail [多納林道], 20.III.2010, leg. U. Ong; Miaoli: 1♀ (NMNS), Hsueshanken [雪山坑], 16-17.III.1995, leg. W. T. Yang; Nantou: 1♀ (NMNS), Howangshan [合望山], 1997, leg. C. C. Lo; 1♀ (NMNS), Huisun Forest Rec. Area [惠蓀林場], 22.V.1997, leg. C.W. & L.B. O’Brien; 2♀♀ (NMNS), Lushan [盧山], 18.V.1997, leg. C. W. & L. B. O’Brien; 1♂ (NMNS), Meifeng [梅峰], 9-10.II.1999, leg. C. S. Lin & W. T. Yang; 2♂♂ (NMNS), Meihsi [眉溪], 16.VI.1965, leg. B. S. Chang; 8♂♂, 4♀♀ (NMNS), Nanshanhsi [南山溪], 21.V.-17.VI.1965, leg. B. S. Chang; 1♂ (NMNS), same locality 11.II.1999, leg. C.-S. Lin; 1♀ (TARI), same locality, 7.IV.2010, leg. Y.-T. Wang; 1♀ (NMNS), Penpuhsi [本部溪], 29.V.1965, leg. B. S. Chang; 2♀♀ (NMNS), same but with “17.V.1970”; 1♀ (NMNS), Shihtzutou [獅子頭], 21.II.1998, leg. C.-C. Lo; 1♂ (TARI), Tungpu [東埔], 19-23.VII.1982, leg. L. Y. Chou & T. Lin; 1♂ (TARI), same locality, 10-14.I.1983, leg. K. C. Chou & S. P. Huang; 4♂♂ (BMNH), Musha [=Wushe, 霧社], 18.V.-15.VI.1919, leg. T. Okuni, J. Sonan, K. Miy., M. Yosh.; 1♂ (TARI), same locality, 19-22.IV.1983, leg. K. C. Chou & S. P. Huang; 1♂, 1♀ (TARI), Yuanfeng [鳶峰], 2.VI.2012, leg. J.-F. Tsai; Pingtung: 1♂ (TARI), Ali [阿禮], 17.II.2016, leg. Y.-T. Chung; 1♂ (TARI), Peitawushan [北大武山], 17.II.2010, leg. S.-F. Yu; 1♂ (TARI), Tahanshan [大漢山], 16.IV.2007, leg. Y.-L. Lin; 1♂ (TARI), same locality, 21.V.2007, leg. Y.-L. Lin; 10♂♂, 2♀♀ (TARI), same locality, 18.VII.2007, leg. C.-F. Lee; 1♂ (TARI), Wutain [霧台], 11.IV.2007, leg. Y.-L. Lin; 1♀ (TARI), same locality, 12.V.2009, leg. U. Ong; Taichung: 2♂♂ (TARI), Kukuan [谷關], 20-22.VI.1978, leg. K. S. Lin & K. C. Chou; Tainan: 1♂ (TARI), Meiling [梅嶺], 4.VI.2010, leg. U. Ong; 1♀ (TARI), same locality, 6.VII.2012, leg. Y.-L. Lin; Taipei: 2♂♂, 2♀♀, Wulai [烏來], 23.I.2008, leg. S.-F. Yu; Taitung: 1♂ (TARI), Chipen [知本], 15-17.II.1981, leg. L. Y. Chou & T. Lin; 1♂ (TARI), Tulanshan [都蘭山], 4.VII.2016, leg. S.-P. Wu; 1♀ (TARI), Yanping trail [延平林道], 5.III.2016, leg. S.-P. Wu; Taoyuan: 1♀ (NMNS), Junghua [榮華], 15.V.1971, leg. B. S. Chang; 2♀♀ (TARI), Paling [巴陵], 3-5.V.1983, leg. K. C. Chou & C. C. Pan.

#### Distribution.

China, Japan, Korea, Russia, Taiwan.

### Gallerucida flaviventris

Taxon classificationAnimaliaORDOFAMILIA

(Baly)

Figs 4A–C , 5

Eustetha flaviventris Baly, 1861: 296.
Galerucida [sic!] (Eusthetha) flaviventris: Weise 1924: 142 (catalogue).
Galerucida [sic!] flaviventris: Ogloblin 1936: 365 (redescription).
Gallerucida flaviventris : Gressitt and Kimoto 1963: 723 (China: Anhui, Jiangsu, Jiangxi, Sichuan, Zhejiang); Kimoto 1969: 68 (Taiwan); Wilcox 1971: 203 (catalogue); Kimoto 1989: 260 (Taiwan); Kimoto 1991: 17 (Taiwan); Kimoto and Chu 1996: 91 (catalogue); Kimoto and Takizawa 1997: 392 (catalogue); Beenen 2010: 459 (catalogue); Yang et al. 2015: 172 (catalogue); Lee et al. 2016: 96 (biology).
Sphenoraia chujoi Lee, 2014: 143. **syn. n.**

#### Type material.

*Eustetha flaviventris*. Lectotype ♀ (BMNH), here designated, labeled: “Type [p, w, circular label with red border] // Baly Coll. [p, w] // Eustetha / flaviventris / Baly / N. China [h, g]”. Number of paralectotypes is uncertain.

*Sphenoraia chujoi*. Holotype ♂ (TARI): “Sôzan [h] [= Yangmingshan, 陽明山] / FORMOSA [p] / 25.X.1936 [h] / COL. M. CHUJO[p, w] // **Holotypus** / *Sphenoraia chujoi* / Lee, sp. nov. / det. C.-F. Lee, 2014 [p, r]”. Paratypes: 3♀♀ (TARI): “Sôzan [h] / FORMOSA [p] / 25.X.1936 [h] / COL. M. CHUJO[p, w] // **Paratypus** / *Sphenoraia chujoi* / Lee, sp. nov. / det. C.-F. Lee, 2014 [p, pink label]”

#### Diagnosis.

*Gallerucida flaviventris* adults are similar to those of *G. shirozui* Chûjô and *G. thoracica* Jacoby in possessing metallic elytra, but are easily recognized by their metallic pronota (yellow brown pronota with black spots in other species).

#### Redescription.

See description of *Sphenoraia chujoi* Lee (2014).

#### Variation.

**Figures 5.**
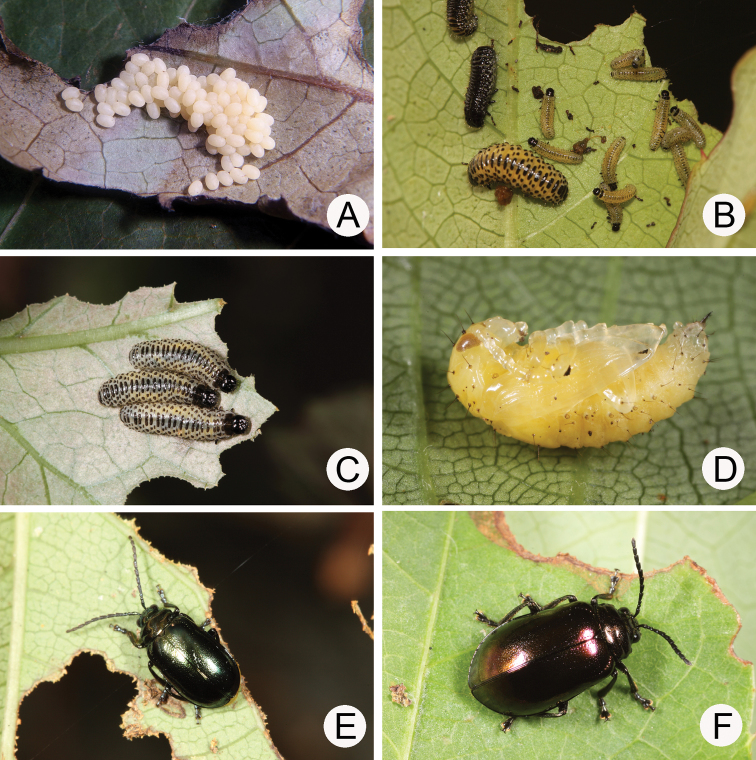
Field photographs of *Gallerucida flaviventris* (Baly). **A** Egg mass **B** Early instar larvae **C** Mature larvae **D** Pupa **E** Adult, typical form **F** Adult, color variation.

Specimens from China are uniformly metallic blue (Fig. 4C) but those from Taiwan are metallic green, bronze, or purple (Figs 4A, B, 5E, F).

**Figures 4.** Habitus of *Gallerucida* species. **A** *G. flaviventris* (Baly), male from Taiwan, dorsal view **B** Ditto, ventral view **C** *G. flaviventris* (Baly), female from China, dorsal view **D** *G. singularis* Harold, male, dorsal view **E** Ditto, ventral view **F** *G. singularis* Harold, female, dorsal view **G** *G. singularis* Harold, posterior view **H** *G. gebieni* Weise, posterior view.

#### Host plants.

Polygonaceae: Polygonatum odoratum Docuce var. pluriflorum Ohwi (Yu et al. 1996); Vitaceae: *Cayratia* sp. (Yu et al. 1996); *Parthenocissus tricuspidata* (Sieb. & Zucc.) Planch. (Lee et al. 2016).

#### Biology.

*Gallerucida flaviventris* populations are presumably univoltine. The following life cycle information is based on our (TCRT) observations (Lee et al. 2016). Females began to deposit an average of 80 eggs in single egg mass (Fig. 5A) during late March. Eggs hatched in 11 days. The larvae (Fig. 5B) fed on leaves and the larval duration was 14 days. Mature larvae (Fig. 5C) burrowed into soil and built underground chambers for pupation. Duration of the pupal stage (Fig. 5D) was 15–18 days. Newly emerged adults appeared during spring and were active (Fig. 5E–5F) during summer and autumn.

#### Remarks.

When Lee (2014) described *Sphenoraia chujoi*, the character of the metasternum was overlooked. This species is certainly attributed to *Gallerucida flaviventris*.

#### Other material examined.

**CHINA**. Anhui: 1♂ (CAS), Tung-Lu, 30.III.1926, leg. D. E. Wright; Fujian: 3♂♂ (TARI), Jiuxianshan [九仙山], 22.VI.2014; 1♂, 2♀♀ (TARI), same locality, 14.VI.2015; Guanxi: 1♂ (TARI), Dayaoshan [大瑤山], 16.IV.2016; Hong Kong: 1♀ (BMNH); Sichuan: 1♂ (CAS), Chang-Tau-Ching, 18.VII.1948, leg. Gressitt & Djou; Zhejiang: 1♂ (BPBM), Hangchow [= Hangzhou, 杭州], 2.VII.1924, leg. J. F. Illingworth; 2♂♂ (1♂: BPBM; 1♂: KMNH), same but with “3.IV.1924; **TAIWAN**. Taipei: 1♂, 2♀♀ (TARI), Lengshuikeng [冷水坑], 4–5.VII.2009, leg. J.-C. Chen; 1♀ (TARI), Mientienshan [面天山], 22.X.2011, leg. M.-H. Tsou; 31 exs., (TARI), Neishuanghsi [內雙溪], reared from eggs, 12-17.V.2010, leg. M.-H. Tsou; 3♂♂, 2♀♀ (TARI), Tatunhsi trail [大屯溪古道], 28.V.2013, leg. H. Lee; 1♀ (TARI), Tatunshan [大屯山], 26.V.2010, leg. S.-F. Yu; 1♂ (TARI), Tienhsiyuan [=天溪園], 8.V.2015, leg. H. Lee; 1♂ (TARI), Yangmingshan [陽明山], 6.X.2008, leg. J.-C. Chen; Taitung: 1♂, 3♀♀ (EUMJ), Luye (鹿野), 8.IV.2012, leg. Yamasako; Taoyuan: 1♂ (TARI), Hsuanhuan [萱源], 13.V.2010, leg. S.-F. Yu.

#### Distribution.

China, Taiwan.

### Gallerucida gebieni

Taxon classificationAnimaliaORDOFAMILIA

Weise

Fig. 4H

Galerucida [sic!] gebieni Weise, 1922: 92; see Lee and Bezděk 2013: 367 for complete list.

#### Diagnosis.

*Gallerucida gebieni* and *G. singularis* Harold adults are easily recognized by their reddish brown bodies and black spots behind humeral calli and elytral apices, but adults of *G. gebieni* possess only two black spots on the elytral apices (Fig. 4H) (three spots in *G. singularis* (Fig. 4G)).

#### Redescription.

See Lee and Bezděk (2013).

#### Host plant.

Polygonaceae: *Polygonum chinense* L. (Aston 2009).

#### Distribution.

China, Taiwan (only in Kinmen and Nankan islands).

### Gallerucida lutea

Taxon classificationAnimaliaORDOFAMILIA

Gressitt & Kimoto

Figs 6A–C , 7 , 8

Gallerucida lutea Gressitt & Kimoto, 1963: 124 (China: Guangdong, Hubei); Kimoto 1969: 68 (Taiwan); Wilcox 1971: 204 (catalogue); Kimoto and Chu 1996: 92 (catalogue); Kimoto and Takizawa 1997: 392 (catalogue); Lee and An 2001: 127 (Korea); Beenen 2010: 459 (catalogue); Lee and Cheng 2010: 90 (biology); Yang et al. 2015: 173 (catalogue).

#### Type material.

Holotype ♂ (CAS), labeled: “N. KWANGTUNG / China, Lochang, [p,w] // 1947 [h, w] // L. Gressitt / Collection [p, w] // HOLOTYPE [p] ♂ / Gallerucida / lutea [h] / Gressitt & Kimoto [p, r] // Gallerucida / lutea / Holo G & K [h] / J. L. Gressitt det. [p, w] // California Academy / of Sciences / Type / No. [p] 13271 [h, w]”. Paratypes: 1♂ (BPBM): “N. KWANGTUNG / China, Lochang, [p,w] // 1947 [h, w] // L. Gressitt / Collection [p, w] // ALLOTYPE [p] / Gallerucida / lutea ♀ [h] / Gressitt & Kimoto [p, r] // 3321 [h, w] // Gallerucida / sp. nov. 6 / lutea. Allo [h] / Det. S. Kimoto [p] 61 [h, w]”; 1♀ (CAS), labeled: “Suisapa, 1000 M. / Lichuen Distr. / W. Hupeh, China / VII-30-48 [p, w] // Gressitt & / Djou Collrs. [p, w] // PARATYPE [p] / Gallerucida / lutea [h] / Gressitt & Kimoto [p, y] // Galerucida / s. p. lutea / (nr. sp.6) [h] / Det. Kimoto [p] ’61 [h, w]”.

#### Diagnosis.

*Gallerucida lutea* adults can be recognized by their yellowish brown bodies. Darker individuals of *G. lutea* may look like entirely black individuals of *G. bifasciata*, but the elytra of *G. lutea* possess extremely coarse punctures and minute punctures between coarse punctures and filiform antenna.

#### Redescription.

**Figures 6.**
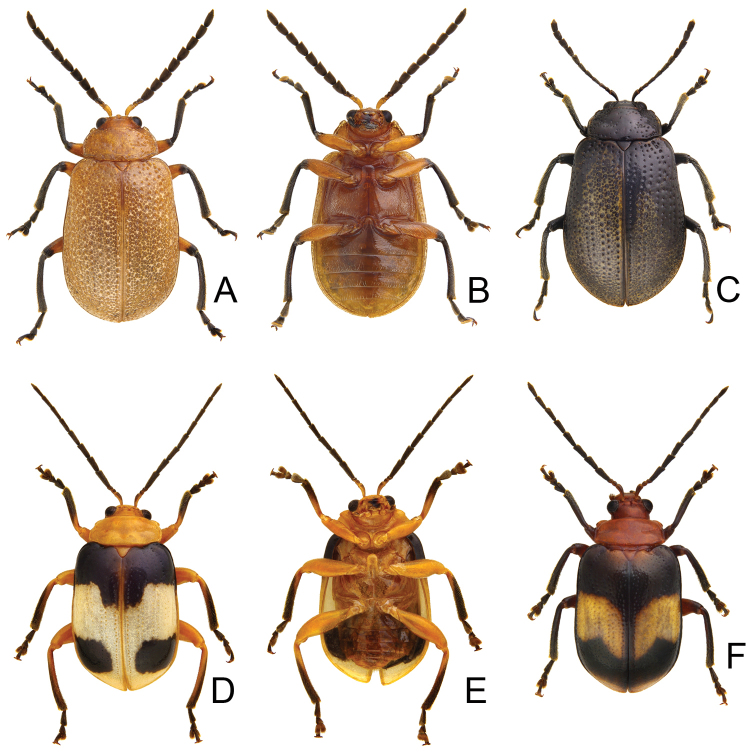
Habitus of *Gallerucida* species. **A** *G. lutea* Gressitt & Kimoto, male, dorsal view **B** Ditto, ventral view **C** *G. lutea* Gressitt & Kimoto, female, color variation, dorsal view **D** *G. sauteri* Chûjô, male, dorsal view **E** Ditto, ventral view **F** *G. sauteri* Chûjô, male, color variation, dorsal view.

**Figures 7.**
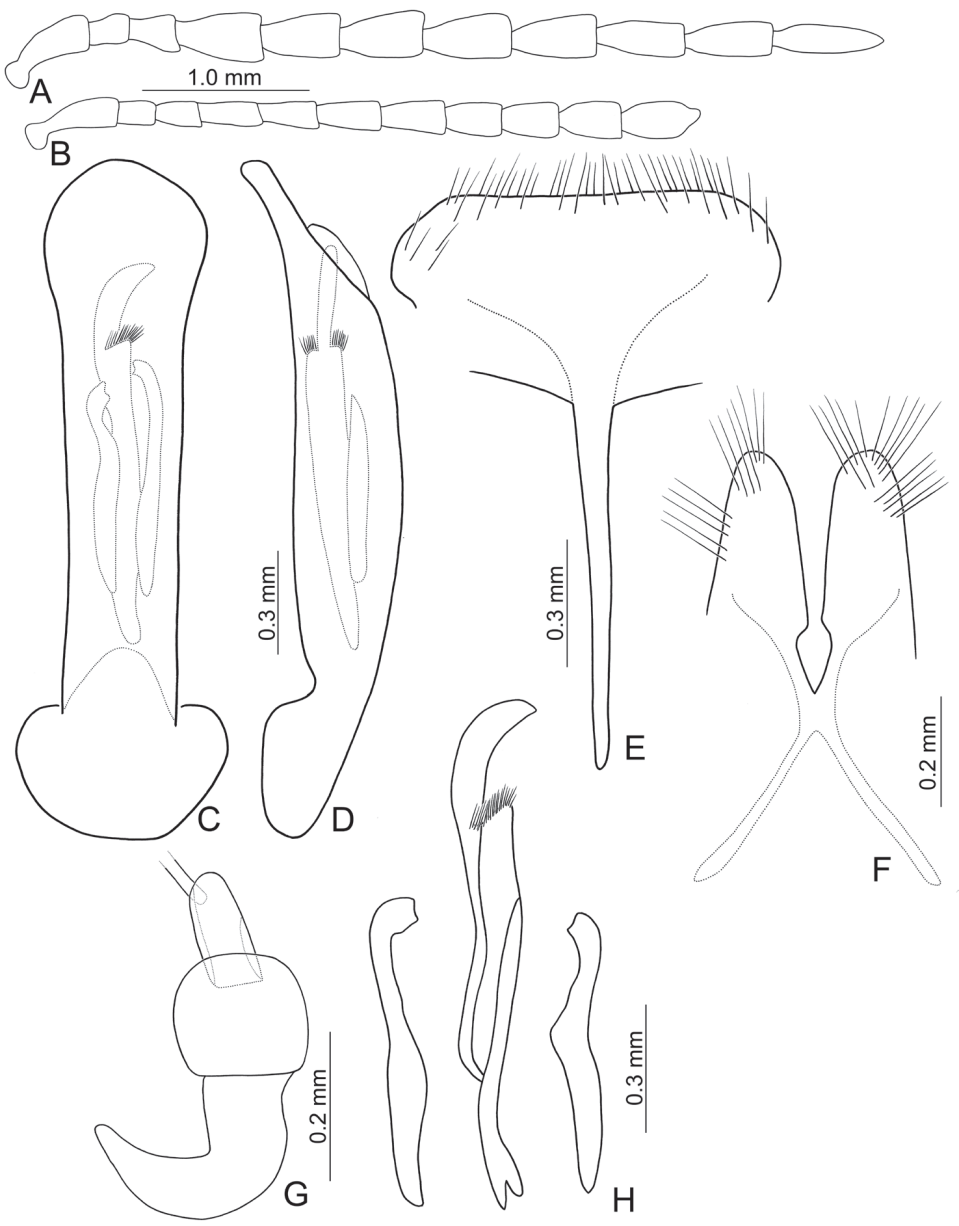
Diagnostic characters of *Gallerucida lutea* Gressitt & Kimoto. **A** Antenna, male **B** Antenna, female **C** Penis, dorsal view **D** Penis, lateral view **E** Abdominal ventrite VIII **F** Gonocoxae **G** Spermatheca **H** Endophallic sclerites.

Length 8.4–9.8 mm, width 4.7–5.9 mm. General color (Fig. 6A–B) yellowish or reddish brown; antenna black except three basal antennomeres; tibiae and tarsi entirely black. Antenna serrate in male (Fig. 7A), length ratios of antennomeres I–XI 1.0 : 0.4 : 0.6 : 0.9 : 0.8 : 0.9 : 0.9 : 0.9 : 0.9 : 0.9 : 1.2, length to width ratios of antennomeres I–IX 2.5 : 1.4 : 1.5 : 1.8 : 1.8 : 1.9 : 2.0 : 2.0 : 2.5 : 2.6 : 4.0; antennomeres IV-VII filiform and VIII-X serrate in female (Fig. 7B), length ratios of antennomeres I–XI 1.0 : 0.4 : 0.5 : 0.7 : 0.6 : 0.7 : 0.7 : 0.6 : 0.6 : 0.6 : 0.8, length to width ratios of antennomeres I–IX 3.1 : 1.5 : 2.0 : 2.6 : 2.2 : 2.1 : 1.8 : 1.6 : 1.6 : 1.7 : 2.2. Pronotum transverse, 1.9× wider than long, disc convex, with oblique depressions at sides, medially abbreviated, disc without microreticulation, with extremely coarse, sparse punctures; lateral margin slightly rounded; apical margin concave; basal margin convex. Elytra parallel from base to basal 1/3, gradually widened towards basal 1/3, lateral margin serrate subapically; 1.4× longer than wide, disc without microreticulation but with extremely coarse punctures arranged into striae, with tiny punctures between strial punctures; dorso-ventrally flattened. Penis (Fig. 7C–D) elongate, 6.4× longer than wide; parallel-sided; abruptly widened from apical 1/3 to 1/6, apex circular; slightly curved at lateral view; ventral surface well sclerotized; endophallic sclerite complex (Fig. 7H) large, about 0.6× as long as penis, composed of one median sclerite and one pair of lateral sclerites, median sclerite longitudinal, strongly curved near apex, with lateral process at apical 1/4, with dense setae along apical margin of lateral process; lateral sclerites longitudinal but much shorter, about 0.5× as long as median one, curved near apex, apices truncate or concave. Gonocoxae (Fig. 7F) wide, connected from base to middle, apices rounded, with dense elongate setae. Ventrite VIII (Fig. 7E) longitudinal, apex transverse, apical margin truncate; with dense short setae along lateral and apical margin; spiculum slender. Receptacle of spermatheca (Fig. 7G) strongly swollen; pump short but strongly curved; proximal spermathecal duct wide and deeply inserted into receptacle.

#### Variation.

Some individuals have black legs and bodies darker than usual (Fig. 6C).

#### Host plant.

Vitaceae: *Vitis kelungensis* Moriyama (Lee and Cheng 2010).

#### Biology.

**Figures 8.**
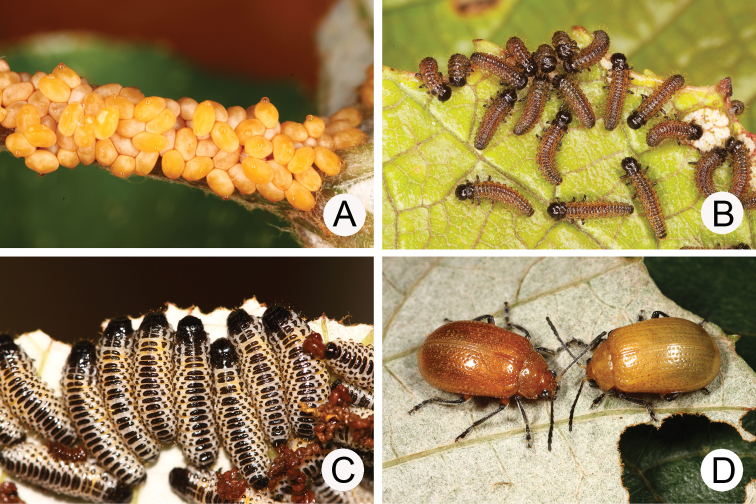
Field photographs of *Gallerucida lutea* Gressitt & Kimoto. **A** Egg mass **B** Early instar larvae **C** Mature larvae **D** Adults.

*Gallerucida lutea* populations are presumably univoltine. The following life cycle information is based on our (TCRT) observations (Lee and Cheng 2010). Females began to deposit an average of 140 eggs in single egg masses (Fig. 8A) during April or May. Eggs hatched in 9 days. The larvae (Fig. 8B) fed on leaves and the larval duration was 11 days. Mature larvae (Fig. 8C) burrowed into the soil and built underground chambers for pupation (fig. 33E). Duration of the pupal stage was 15–17 days. Newly emerged adults appeared during spring and were active (Fig. 8D) during summer and autumn.

#### Other material examined.

**CHINA**. 3♂♂, 3♀♀ (BMNH); **TAIWAN**. Kaoshiang: 1♀ (TARI), Tona trail [多納林道], 3.XII.2012, leg. W.-C. Liao; 1♂ (TARI), same locality, 10.IX.2014, leg. B.-X. Guo; Keelung: 1♀ (TARI), Kangtzuliao [槓子寮], 28.IX.2011, leg. H. Lee; Taipei: 1♂ (TARI), Yangmingshan [陽明山], 15.III.1998, leg. C.-F. Lee; 7♂♂, 11♀♀ (TARI), same locality, reared from eggs, 6.VII.2008, leg. M.-H. Tsou; 11♂♂, 10♀♀ (TARI), same locality, reared from eggs, 26.V.2009, leg. M.-H. Tsou; 1♂ (TARI), Yulu trail [魚路古道], 6.VII.2008, leg. M.-H. Tsou; 1♀ (TARI), same but with “3.V.2009”.

#### Distribution.

China, Korea, Taiwan.

### Gallerucida sauteri

Taxon classificationAnimaliaORDOFAMILIA

Chûjô

**Figures 9.**
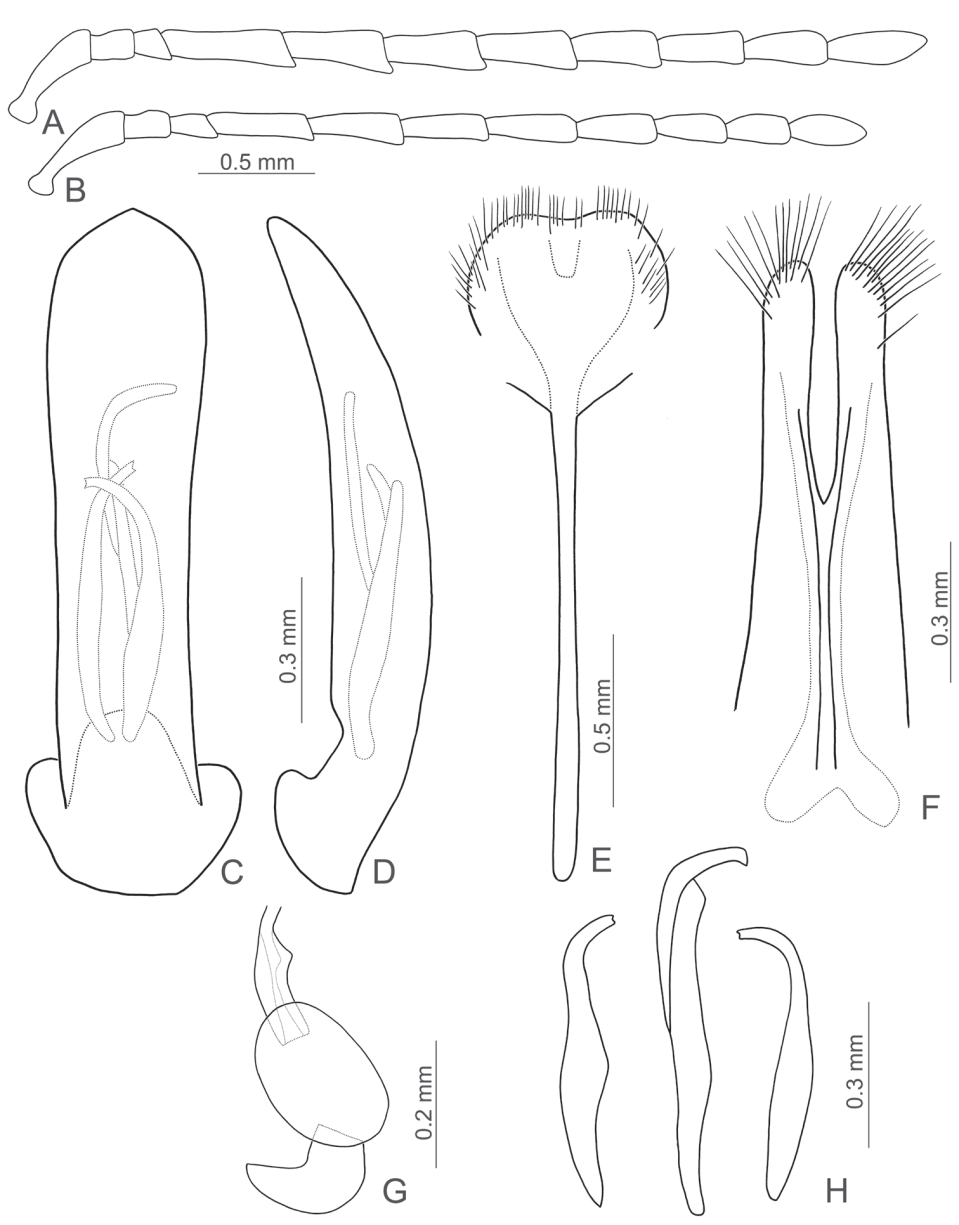
Diagnostic characters of *Gallerucida sauteri* Chûjô. **A** Antenna, male **B** Antenna, female **C** Penis, dorsal view **D** Penis, lateral view **E** Abdominal ventrite VIII **F** Gonocoxae **G** Spermatheca **H** Endophallic sclerites.

**Figure 10.**
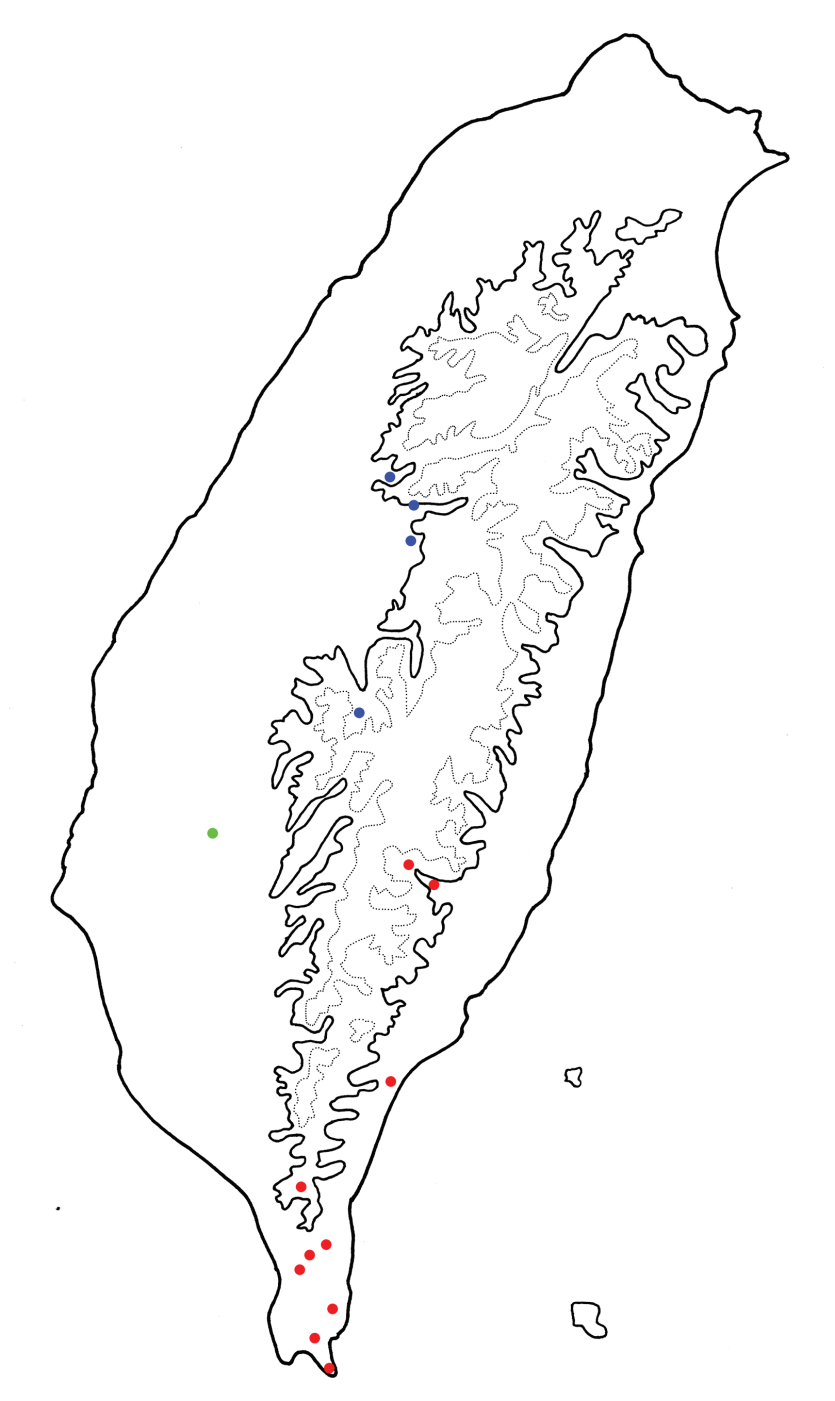
Distribution map of *Gallerucida sauteri* Chûjô, solid line: 1000 m, broken line: 2000m. **Red dots** Typical form **Blue dots** Color variation as *G. quadraticollis* **Green dot** Intermediate form.

**Figures 11.**
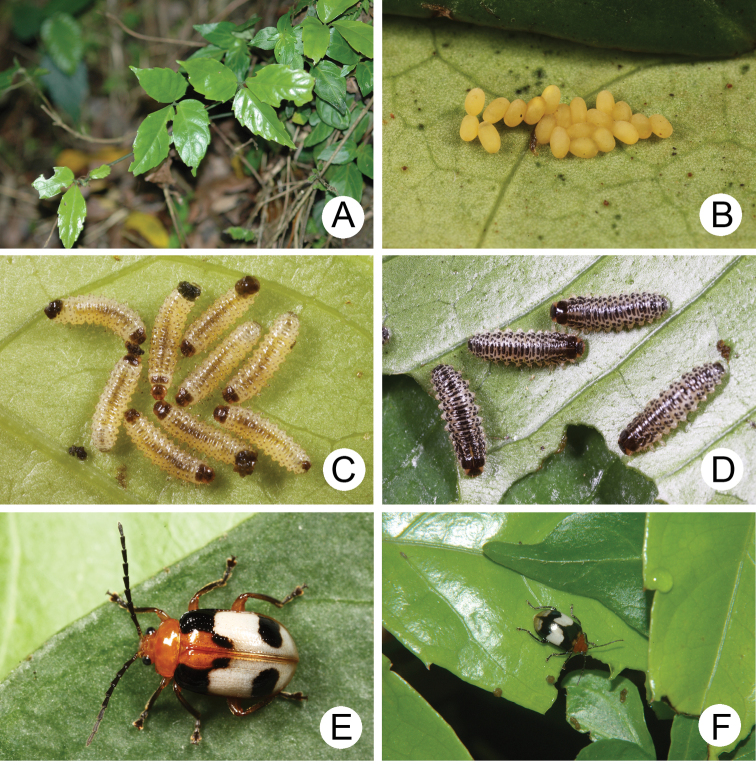
Field photographs of *Gallerucida sauteri* Chûjô. **A** Host plant: *Tetrastigma formosanum* **B** Egg mass **C** Early instar larvae **D** Mature larvae **E** Adult, typical form **F** Adult, color variation.

Figs 6D–F , 9 , 10 , 11

Gallerucida sauteri Chûjô, 1938: 141; Chûjô 1962: 152 (redescription); Kimoto 1966: 35 (Taiwan); Wilcox 1971: 206 (catalogue); Kimoto and Chu 1996: 92 (catalogue); Kimoto and Takizawa 1997: 392 (catalogue); Beenen 2010: 460 (catalogue); Lee and Cheng 2010, 92 (biology); Yang et al. 2015: 176 (catalogue).
Gallerucida quadraticollis Takizawa, 1978: 127; Kimoto and Chu 1996: 92 (as synonym of G. sauteri, synonym confirmed).

#### Type material.

*Gallerucida sauteri*. Lectotype ♂ (TARI), here designated, labeled: “Kankau (Koshun [= Henchu, 恆春]) / Formosa / H. Sauter V. 1912 [p, w] // CO / Type [p, w, yellow letters, circular label with yellow border] // Galerucida / sauteri / Chûjô [h] / M. CHUJO [p, g] // 1936 [p, w]”. Paralectotypes. 1♂ (TARI), same as lectotype but with “1368”; 1♂ (SDEI): “Kankau (Koshun) / Formosa / H. Sauter V. 1912 [p, w] // Syntypus [p, r] // Galerucida / sauteri / Chûjô [h] / M. CHUJO [p, g] // DEI Müncheberg / Col-09173 [p, g]”; 1♂ (SDEI): “VIII [h] Koshun / Formosa / H. Sauter [p] 18 [h, w] // Syntypus [p, r] // Galerucida / sauteri / Chûjô [h] / M. CHUJO [p, g] // DEI Müncheberg / Col-09172[p, g]”; 1♀ (TARI): “Formosa. / Taito [= Taitung, 台東], 1919. / II 25-III 27. / S. Inamura [p, w] // CO / Type [p, w, yellow letters, circular label with yellow border] // Galerucida / sauteri / Chûjô [h] / M. CHUJO [p, g]”; 1♀ (TARI): “CHIPON [h] [= Chihpen, 知本] / FORMOSA [p] / 25.III.1935 [h] / COL. M. CHUJO [p, w] // CO / Type [p, w, yellow letters, circular label with yellow border] // Galerucida / sauteri / Chûjô [h] / M. CHUJO [p, g] // No. 1358 [p, w]”.

*Gallerucida quadraticollis*. Holotype ♂ (EIHU): “Tungpu [東埔] / Chiayi Taiwan / 14-17.VII.1976 / H. Takizawa [p, w] // Holo [h] type [p] / Gallerucida / quadraticollis / Takizawa [h, r] // HOLOTYPE / Appended label by ÔHARA, IMRAI, KANBE / SUZUKI and HIRONAGA / 2007 [p, w, with red band along right margin] // 0000003056 / Sys. Ent / Hokkaido Univ. / Japan [SEHU] [p, w]”.

#### Diagnosis.

*Gallerucida sauteri* adults may be recognized by the white elytra possessing black transverse stripes.

#### Redescription.

Length 5.8–7.8 mm, width 3.3–4.3 mm. General color (Fig. 6D–E) yellowish brown; antenna black except three basal antennomeres; elytra pale yellow or white, with wide transverse black band from base to basal 1/4, extending posterior at middle and truncate; sometimes median area of base reddish brown (Fig. 11E), with one pair of transverse black bands at apical 1/3, interrupted by suture; legs yellow but tibiae and tarsi partly or entirely dark brown to black. Antenna slightly serrate in male (Fig. 9A), length ratios of antennomeres I–XI 1.0 : 0.4 : 0.3 : 1.2 : 1.0 : 1.0 : 0.9 : 0.8 : 0.8 : 0.8 : 1.0, length to width ratios of antennomeres I–IX 3.2 : 1.4 : 1.2 : 3.5 : 2.4 : 2.6 : 2.5 : 2.2 : 2.5 : 2.5 : 3.3; filiform and shorter in female (Fig. 9B), length ratios of antennomeres I–XI 1.0 : 0.4 : 0.4 : 0.9 : 0.8 : 0.8 : 0.8 : 0.7 : 0.6 : 0.6 : 0.7, length to width ratios of antennomeres I–IX 3.3 : 1.6 : 2.1 : 3.1 : 3.2 : 3.1 : 3.2 : 2.8 : 2.6 : 2.3 : 2.6. Pronotum transverse, 2.1× wider than long, disc convex, with oblique depressions at sides, medially abbreviated, disc with micro-reticulation but lacking punctures; lateral margin straight or slightly rounded; apical margin concave; basal margin convex. Elytra parallel from base to basal 1/3, gradually widened towards basal 1/3; 1.4× longer than wide, disc without micro-reticulation but with coarse punctures; dorso-ventrally flattened. Penis (Fig. 9C–D) elongate, 5.2× longer than wide; parallel-sided; apex widely lanceolate; curved at lateral view; ventral surface well sclerotized; endophallic sclerite complex (Fig. 9H) large, about 0.5× as long as penis, composed of one median sclerite and one pair of lateral sclerites, median sclerite longitudinal, strongly curved near apex, lateral sclerites longitudinal but slightly shorter, about 0.8× as long as median sclerite, strongly and apically curved, apices truncate or concave. Gonocoxae (Fig. 9F) elongate, connected from near base to basal 3/5, apices rounded, with dense long setae; base wide. Ventrite VIII (Fig. 9E) longitudinal, apical margin truncate but medially depressed; with dense short setae along lateral and apical margin; spiculum extremely slender. Receptacle of spermatheca (Fig. 9G) strongly swollen; pump short but strongly curved; proximal spermathecal duct slender and deeply inserted into receptacle.

#### Variation.

The typical adult color pattern occurs in southern Taiwan (Fig. 10). Populations in central Taiwan have a black band at the elytral base extending posterior and acute apically; black spots at apices well developed, widened and connected with each other. The latter forms were described as *G. quadraticollis* by Takizawa (1978) (Figs 6F, 11F). Intermediate individuals were collected from Meiling [梅嶺] having anterior spots at the elytra similar to the typical form but posterior ones similar those of *G. quadraticollis*.

#### Host plants.

Vitaceae: *Tetrastigma formosanum* (Hemsl.) Gagnep (Fig. 11A) (Lee and Cheng 2010).

#### Biology.

*Gallerucida sauteri* populations are presumably multivoltine. The following life cycle information is based on our (TCRT) observations (Lee and Cheng 2010). Females began to deposit an average of 20 eggs in single egg masses (Fig. 11B) during late March. Eggs hatched in seven days. The larvae (Fig. 11C) fed on leaves and the larval duration was 13 days. Mature larvae (Fig. 11D) burrowed into soil and built underground chambers for pupation. Duration of the pupal stage was 10–11 days. Newly emerged adults appeared during spring and were active (Fig. 11E, 11F) during summer and autumn.

#### Other material examined.

**TAIWAN.** Nantou: 1♀ (NMNS), Lienhuachih [蓮華池], 9.IV.-2.V.2001, leg. C, S, Lin & W. T. Yang; 1♀ (NMNS), same but with “12.VI.-19.VII.2001”; 1♀ (NMNS), same but with “17.X.-14.XI.2001”; 1♀ (NMNS), same but with “1.VIII.-7.IX.2005”; 1♂ (TARI), Tungpu [東埔], 23-27.VII.1984, leg. K. C. Chou & C. H. Yang; Pingtung: 1♀ (TARI), Lilungshan [里龍山], 5.XI.2009, leg. M.-H. Tsou; 1♀ (TARI), Nanjenshan [南仁山], 1.III.2010, leg. J.-L. Jeng; 1♀ (TARI), Ouluanpi [鵝鑾鼻], 24.II.1982, leg. T. Lin & S. C. Lin; 2♂♂, 1♀ (TARI), Sheting [社頂], 15.VIII.2009, leg. M.-H. Tsou; 1♂ (TARI), same locality, 17.VIII.2010, leg. J.-C. Chen; 1♀ (TARI), Shouka [壽卡], 23.II.2013, leg. W.-C. Liao; 1♂, 3♀♀ (TARI), Tahanshan [大漢山], 20.VII.2007, leg. S.-F. Yu; 3♂♂, 3♀♀ (TARI), same but with “leg. C.-F. Lee”; 2♂♂ (TARI), same locality, 15.XII.2015, leg. W.-C. Liao; Taichung: 1♂ (TARI), Wushihkeng [烏石坑], 13.VII.2008, leg. C.-F. Lee; 98 exs. (TARI), same locality, 15-19.V.2013, leg. C.-F. Lee; Tainan: 3♂♂ (TARI), Meiling [梅嶺], 12.III.2011, leg. M. L. Jeng; Taitung: 2♂♂ (TARI), Chinlun trail [金崙林道], 11.I.2016, leg. J.-C. Chen; 1♂ (TARI), Liyuan [栗園], 19.VI.2013, leg. B.-X. Guo; 1♀ (TARI), Tienlung trail [天龍古道], 20.III.2015, leg. J.-C. Chen.

#### Distribution.

Endemic to Taiwan.

### Gallerucida shirozui

Taxon classificationAnimaliaORDOFAMILIA

Kimoto

Figs 12A–C , 13

Gallerucida shirozui Kimoto, 1969: 67 (Taiwan); Wilcox 1971: 206 (catalogue); Kimoto and Chu 1996: 92 (catalogue); Kimoto and Takizawa 1997: 392 (catalogue); Beenen 2010: 460 (catalogue); Yang et al. 2015: 176 (catalogue).

#### Type material.

Holotype ♂ (KUEC): “(Taiwan) / Sungkang / Nantou Hsien [p, w] // 1.VI. [h] 1965 / T. Shirôzu [p, w] // Gallerucida / shirozui / Kimoto, n. sp. [h, w] // HOLOTYPE [p, r]”.

#### Diagnosis.

*Gallerucida shirozui* and *G. thoracica* Jacoby adults are easily recognized by their metallic elytra and reddish or yellowish brown pronota. Adults of *Gallerucida shirozui* differ from those of *G. thoracica* by possessing only one pair of black spots on the pronotum (two pairs in *G. thoracica*) and longer and more serrate antennae (shorter and filiform antennae in *G. thoracica*).

#### Redescription.

**Figures 12.**
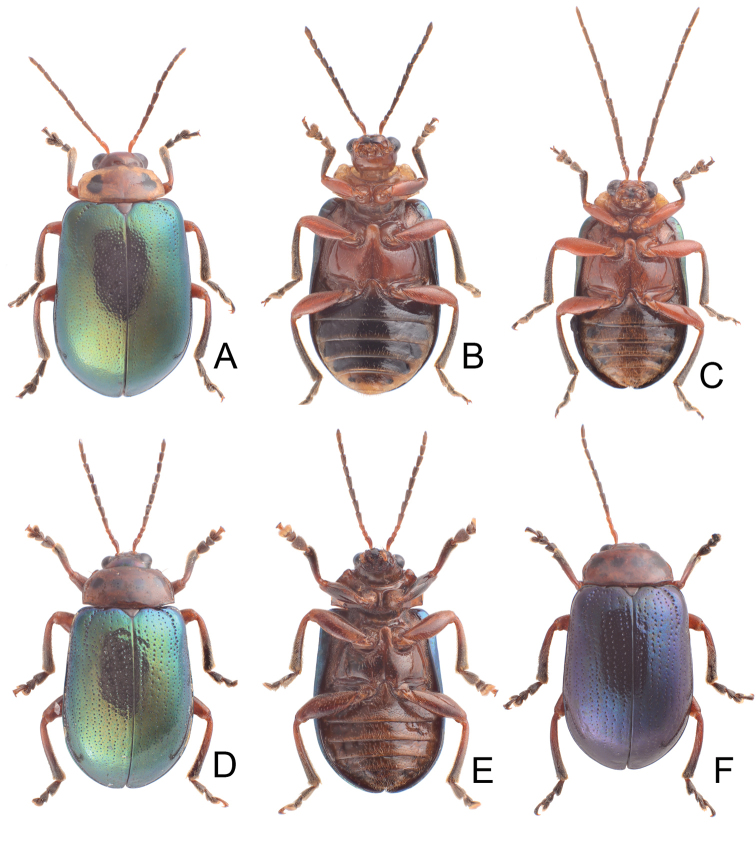
Habitus of *Gallerucida* species. **A** *G. shirozui* Kimoto, female, dorsal view **B** Ditto, ventral view **C** *G. shirozui* Kimoto, male, color variation, ventral view **D** *G. thoracica* (Jacoby), male, dorsal view **E** Ditto, ventral view **F** *G. thoracica* (Jacoby), male, color variation, dorsal view.

**Figures 13.**
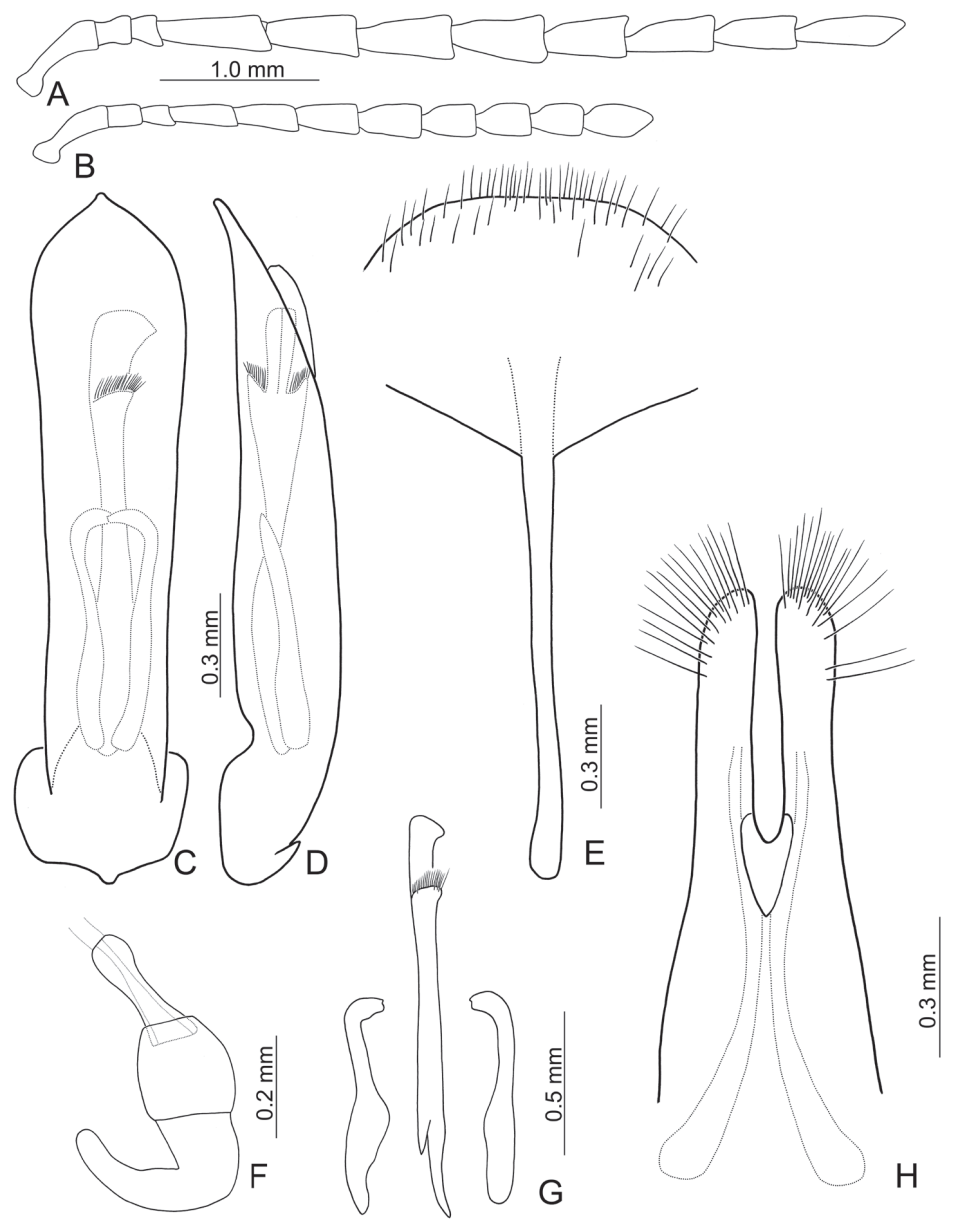
Diagnostic characters of *Gallerucida shirozui* Kimoto. **A** Antenna, male **B** Antenna, female **C** Penis, dorsal view **D** Penis, lateral view **E** Abdominal ventrite VIII **F** Spermatheca **G** Endophallic sclerites **H** Gonocoxae.

Length 7.2–8.2 mm, width 3.8–5.2 mm. General color (Fig. 12A–C) reddish brown; antenna black except three basal antennomeres; pronotum yellowish brown with one pair of black spots at sides, brown between black spots; elytra entirely metallic green; tibiae, and tarsi black; each abdominal ventrite with one pair of black spots at sides, sometimes expanding inwards and connected medially. Antenna serrate in males (Fig. 13A), length ratios of antennomeres I–XI 1.0 : 0.4 : 0.4 : 1.2 : 1.0 : 1.0 : 1.0 : 0.9 : 0.9 : 0.9 : 1.1, length to width ratios of antennomeres I–IX 3.2 : 1.2 : 1.1 : 3.3 : 2.2 : 2.2 : 1.9 : 1.9 : 2.4 : 2.4 : 3.9; filiform and much shorter in females (Fig. 13B), length ratios of antennomeres I–XI 1.0 : 0.4 : 0.4 : 0.8 : 0.8 : 0.7 : 0.7 : 0.7 : 0.6 : 0.6 : 0.8, length to width ratios of antennomeres I–IX 3.4 : 1.5 : 1.6 : 3.2 : 2.6 : 2.0 : 1.8 : 1.9 : 1.7 : 1.7 : 2.0. Pronotum transverse, 2.0× wider than long, disc convex, with oblique depressions at sides, medially abbreviated, disc with micro-reticulation and dense, coarse punctures; lateral margin straight or slightly rounded; apical margin concave; basal margin convex. Elytra parallel-sided; 1.4-1.6× longer than wide, disc without micro-reticulation but with dense, coarse punctures arranged randomly; dorso-ventrally flattened. Penis (Fig. 13C–D) elongate, 5.2× longer than wide; parallel-sided; apex widely lanceolate; straight but apically curved in lateral view; ventral surface well sclerotized; endophallic sclerite complex (Fig. 13G) large, about 0.6× as long as penis, composed of one median sclerite and one pair of lateral sclerites, median sclerite longitudinal, straight in lateral view, with dorsal processes at apical 1/5, with dense setae along apical margin of process, lateral sclerites longitudinal but much shorter, about 0.5× as long as median sclerite, strongly curved near apex, apices concave. Gonocoxae (Fig. 13H) elongate, connected from base to basal 3/5, apices rounded, with dense elongate setae; base wide. Ventrite VIII (Fig. 13E) longitudinal, apical margin truncate but laterally membranous; with sparse short setae along and inside apical margin; spiculum extremely slender. Receptacle of spermatheca (Fig. 13F) strongly swollen; pump short but strongly curved; proximal spermathecal duct slender and shallowly inserted into receptacle.

#### Variation.

Females from southern Taiwan possess narrower antennae (length to width ratios of antennomeres I–IX 3.4 : 1.4 : 1.7 : 3.3 : 3.1 : 2.3 : 1.9 : 2.0 : 2.0 : 2.0 : 2.7) and reduced punctures on the pronota.

#### Host plant.

Vitaceae: *Vitis flexuosa* Thunb. (present study).

#### Biology.

Two mature larvae were collected on leaves of *Vitis flexuosa* in Meifeng during late June 2012. They burrowed into the soil shortly after collection and built underground chambers for pupation. Duration of the pupal stage was 25–28 days. Newly emerged adults were entirely yellow, and required three weeks to change color.

#### Other material examined.

**TAIWAN**. Kaoshiung: 1♂ (TARI), Shihshan trail [石山林道], 19-24.XI.2008, leg. C.-T. Yao; 1♀ (TARI), Tengchih [藤枝], 30.III.2009, leg. C.-T. Yao; 3♂♂ (BMNH), Tona trail [多納林道], 25.VII.2017, leg. B.-X. Guo; 1♂, 3♀♀ (TARI), same but with “2.VIII.2017”; Nantou: 1♂ (TARI), Meifeng [梅峰], 20.IV.2011, leg. T.-H. Lee; 1♂, 1♀ (TARI), same locality, reared from larvae, 29.VII.2012, leg. C.-F. Lee; Pingtung: 1♀ (TARI), Wutai [霧台], 18.III.2010, leg. J.-C. Chen.

#### Distribution.

Endemic to Taiwan.

### Gallerucida singularis

Taxon classificationAnimaliaORDOFAMILIA

Harold

Fig. 4D–G

Galerucida [sic!] singularis Harold, 1880: 146; see Lee and Bezděk 2013: 359 for complete list.

#### Diagnosis.

*Gallerucida gebieni* and *G. singularis* Harold adults are easily recognized by their reddish brown bodies and black spots behind the humeral calli and at the elytral apices (Fig. 4D–F) but those of *G. singularis* possess three black spots on the elytra apices (Fig. 4G) (two spots in *G. gebieni* (Fig. 4H)).

#### Redescription.

See Lee and Bezděk (2013).

#### Host plant.

Polygonaceae: *Polygonum chinense* L. and Fallopia multiflora var. hypoleucum (Ohwi) Yonek. et H. Ohashi (Lee and Bezděk 2013).

#### Biology.

*Gallerucida singularis* populations are presumably multivoltine. Adults are found throughout the year. The natural history was described by Lee and Bezděk (2013).

#### Distribution.

China, Taiwan (only in Kinmen and Nankan islands).

### Gallerucida thoracica

Taxon classificationAnimaliaORDOFAMILIA

(Jacoby)

**Figures 14.**
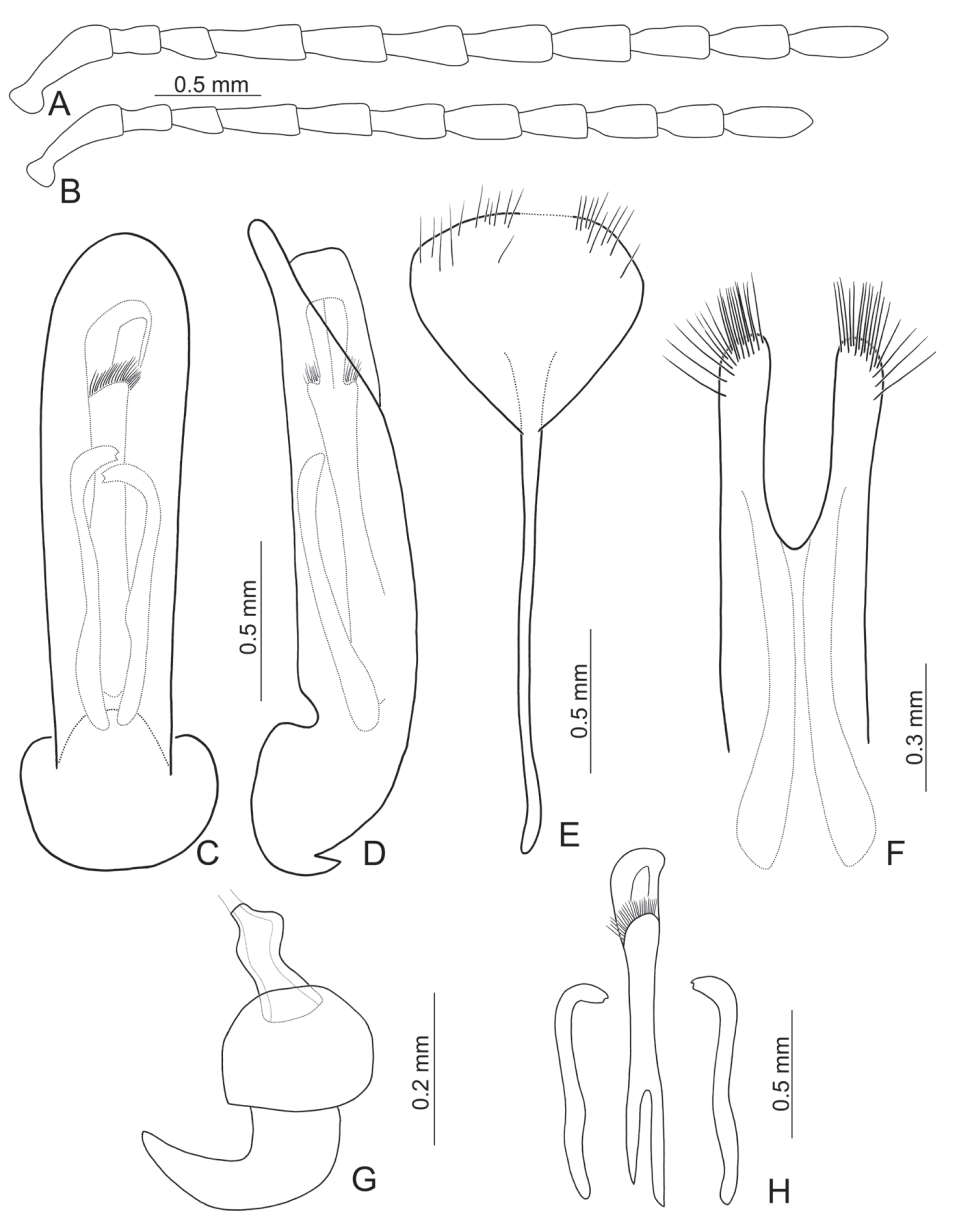
Diagnostic characters of *Gallerucida thoracica* (Jacoby). **A** Antenna, male **B** Antenna, female **C** Penis, dorsal view **D** Penis, lateral view **E** Abdominal ventrite VIII **F** Gonocoxae **G** Spermatheca **H** Endophallic sclerites.

**Figures 15.**
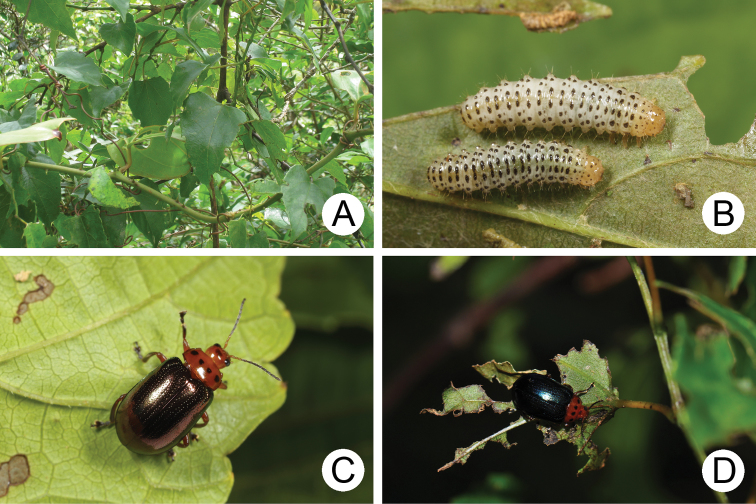
Field photographs of *Gallerucida thoracica* (Jacoby). **A** Host plant: *Vitis flexuosa* **B** Larvae **C** Adult, metallic bronze form **D** Adult, metallic blue form.

Figs 12D–F , 14 , 15

Eustetha thoracica Jacoby, 1888: 348 (China: Jiangxi); Jacoby 1890: 193 (China: Chang-Yang).
Galerucida [sic!] (Eusthetha) thoracica: Weise 1924: 142 (catalogue).
Galerucida [sic!] thoracica: Ogloblin 1936: 362 (redescription).
Gallerucida thoracica : Gressitt and Kimoto 1963: 734 (China); Wilcox 1971: 207 (catalogue); Beenen 2010: 460 (catalogue); Yang et al. 2015: 177 (catalogue).

#### Type material.

Lectotype ♂ (MCZC), here designated, labeled: “Kiukiang / China [h, w] // 1st Jacoby / Coll. [p, w] // *Eustetha* / *thoracica* / Jac. [h, b] // Type [p] / 18241 [h, r]”. Number of paralectotypes is uncertain.

#### Diagnosis.

See diagnosis of *G. shirozui*.

#### Redescription.

Length 7.0–8.9 mm, width 3.8–5.0 mm. General color (Figs 12D–F, 15C, 15D) yellowish brown or reddish brown; antenna black except three basal antennomeres; vertex with one black spot; pronotum with two pairs of black spots at one transverse line; elytra entirely metallic green, or blue, or purple, apical halves of tibiae, and tarsi darker. Antenna filiform in males (Fig. 14A), length ratios of antennomeres I–XI 1.0 : 0.4 : 0.5 : 0.8 : 0.7 : 0.7 : 0.7 : 0.6 : 0.7 : 0.7 : 0.6, length to width ratios of antennomeres I–IX 3.3 : 1.7 : 1.8 : 2.6 : 2.3 : 2.1 : 2.4 : 2.1 : 2.4 : 2.4 : 3.2; shorter in females (Fig. 14B), length ratios of antennomeres I–XI 1.0 : 0.4 : 0.5 : 0.7 : 0.6 : 0.6 : 0.6 : 0.6 : 0.6 : 0.6 : 0.8, length to width ratios of antennomeres I–IX 3.5 : 1.8 : 2.0 : 2.6 : 2.3 : 2.2 : 2.2 : 2.0 : 2.1 : 2.0 : 2.9. Pronotum transverse, 2.1× wider than long, disc convex, with oblique depressions at sides, medially abbreviated, disc with micro-reticulation but lacking punctures; lateral margin straight or slightly rounded; apical margin concave; basal margin convex. Elytra parallel-sided; 1.5× longer than wide, disc without micro-reticulation but with coarse punctures arranged into longitudinal striae, and minute punctures between strial punctures; dorso-ventrally flattened. Penis (Fig. 14C–D) elongate, 5.0× longer than wide; parallel-sided; apex rounded; subapically curved in lateral view; ventral surface well sclerotized; endophallic sclerite complex (Fig. 14H) large, about 0.7× as long as penis, composed of one median sclerite and one pair of lateral sclerites, median sclerite longitudinal, straight in lateral view, with dorsal processes at apical 1/5, with dense setae along apical margin of process, lateral sclerites longitudinal but much shorter, about 0.6× as long as median sclerite, curved near apex, apices concave. Gonocoxae (Fig. 14F) elongate, connected from base to basal 3/5, apices rounded, with dense elongate setae; base wide. Ventrite VIII (Fig. 14E) longitudinal, apical margin truncate but medially membranous; with dense short setae along apical margin, medially abrreviated; spiculum extremely slender. Receptacle of spermatheca (Fig. 14G) strongly swollen; pump short but strongly curved; proximal spermathecal duct slender and deeply inserted into receptacle.

#### Variation.

Chinese specimens possess metallic blue meso- and metathoracic ventrites and legs, and the punctures on the elytra are confused.

#### Host plant.

Vitaceae: *Vitis flexuosa* Thunb. (Fig. 15A) (present study).

#### Biology.

Larvae and adults (Fig. 15B–D) were found on leaves of *Vitis flexuosa* by Ms. Yi-Xuan Hsieh in Tahanshan during early June, 2013. The larvae were transferred to the laboratory for rearing and proved to be *G. thoracica*.

#### Other material examined.

**CHINA**. 1♂ (BPBM), leg. S. V. Mell. **TAIWAN**. Pingtung: 1♀ (TARI), Lilungshan [里龍山], 11.XI.2014, leg. J.-C. Chen; 1♂, 1♀ (TARI), Tahanshan [大漢山], 3.VI.2012, leg. W.-C. Liao; 3♂♂ (TARI), same locality, 6.VII.2012, leg. C.-F. Lee; 1♀ (TARI), same locality, 17.VI.2012, leg. Y.-X. Hsieh; 5♂♂, 3♀♂ (TARI), 4♂♂ (BMNH), same locality, reared from larvae, 26.VI.-8.VII.2012, leg. C.-F. Lee; 2♀♀ (TARI), same locality, 4.VII.2012, leg. M.-H. Tsou; 2♂♂ (TARI), same locality, 20.VII.2013, leg. S.-F. Yu.

#### Distribution.

China, southern Taiwan (new record).

##### Key to Taiwanese species of genus *Gallerucida* Motschulsky

**Table:** 

1	Elytra metallic blue, green or purple	**2**
–	Elytra black, yellowish or reddish brown, or white, sometimes with irregular transverse bands	**4**
2	Pronotum entirely metallic blue, green, or purple (Fig. 4A)	***G. flaviventris***
–	Pronotum yellowish brown, with one or two pairs of black spots	**3**
3	Pronotum with one pair of black spots (Fig. 14A)	***G. shirozui***
–	Pronotum with two pairs of black spots (Fig. 12D, F)	***G. thoracica***
4	Elytra reddish brown, with black spots behind humeral calli and at apices	**5**
–	Elytra black, yellowish brown, or white; sometime with transverse stripes	**6**
5	Two pairs of black spots at elytral apices (Fig. 4H)	***G. gebieni***
–	Three pairs of black spots at elytral apices (Fig. 4G)	***G. singularis***
6	General color yellowish brown, elytra with extremely coarse punctures (Fig. 6A)	***G. lutea***
–	General color black or white, with transverse stripes; elytra with moderately coarse punctures	**7**
7	Elytra black, with three transverse orange stripes, sometimes extremely well developed or completely reduced (Fig. 1)	***G. bifasciata***
–	Elytra white, with two transverse black stripes (Fig. 6D, F)	***G. sauteri***

## Supplementary Material

XML Treatment for Gallerucida bifasciata

XML Treatment for Gallerucida flaviventris

XML Treatment for Gallerucida gebieni

XML Treatment for Gallerucida lutea

XML Treatment for Gallerucida sauteri

XML Treatment for Gallerucida shirozui

XML Treatment for Gallerucida singularis

XML Treatment for Gallerucida thoracica
